# The Problem with Low-Prevalence of Bullying

**DOI:** 10.3390/ijerph15071535

**Published:** 2018-07-20

**Authors:** Arsaell Arnarsson, Thoroddur Bjarnason

**Affiliations:** 1 School of Education, University of Iceland, IS-101 Reykjavik, Iceland; 2 School of Humanities and Social Sciences, University of Akureyri, IS-600 Akureyri, Iceland; thorodd@unak.is

**Keywords:** stress, bullying, life satisfaction, adolescents

## Abstract

This paper tests the hypothesis of whether being bullied in an environment where bullying is infrequent decreases adolescents’ life satisfaction. Analyses were based on the international standard questionnaire from the 2005/2006 Health Behaviour in School-aged Children study (HBSC). The sample included answers from 183,736 children in 35 Western, industrialized countries. Our results show that the negative effects of being bullied on the life satisfaction of individuals are stronger in schools and countries where bullying is less frequent. We therefore conclude that the prevalence of bullying in the students’ surroundings may act as a mediating variable explaining the relationship between bullying and life satisfaction.

## 1. Introduction

Bullying can be defined as physical or verbal aggression that is intentional and repeated. In addition, it involves unequal power between the bully and the bullied and causes distress for the latter [1]. The victims of bullying often suffer a wide range of negative consequences, such as psychological stress [2], psychosomatic illness [3], poor physical health [4], severe depression, suicide ideation [5], poorer psychosocial adjustment [6], substance abuse [7], and social isolation [8] that may persist into adulthood [9]. Bullying in school affects academic achievement, prosocial skills and psychological well-being for both victims and bullies [10,11,12].

Studies have shown that being a victim of bullying significantly reduces the life satisfaction of adolescent students [13,14,15]. Studies have shown that life satisfaction is a key mental health variable during adolescence and an important indicator of individual psychological development [16,17,18]. Furthermore, changes in life satisfaction have been shown to precede changes in psychological health and functioning [19]. Life satisfaction as measured by Cantril’s [20] ladder from the worst possible life to the best possible life is an efficient, global measure with high construct validity across diverse socio-demographic factors such as age, gender, height, income, marital status, employment and religion e.g., [21,22,23]. This measure continues to be routinely used as a measure of quality of life in diverse studies of health-related outcomes e.g., [21,24]. Studies in several countries have demonstrated that Cantril’s [20] measure of life satisfaction is associated with various health behaviours among children, including binge drinking in Wales [25], addictive behaviours in the Netherlands [26], food poverty in Ireland [27], and lack of physical activity and screen-based media use in Canada and the United States [28]. The increasing emphasis on positive psychology has boosted its application. Although the measurements of life satisfaction and psychopathology are related, they are conceptually different. The former defines mental health more in the spirit of positive psychology—as something more than just the absence of pathological symptoms [13].

Studies have firmly established that the prevalence of bully victimization varies significantly by gender [29,30], age [31], race [32], sexual orientation [33], disability [34], family structure [35], family affluence [36] and other factors. Furthermore, the prevalence of victimization varies dramatically between countries [36]. For example, the difference in prevalence of 11-year old children that have been bullied in Lithuania and Armenia is tenfold—30% vs. 3%.

Due et al.’s [3] international comparison study found bully victimization to be associated with psychological symptoms for adolescents in 28 countries in Europe and North America. However, it found that average levels of bullying did not explain between-country differences in symptom prevalence, i.e., high prevalence of bullying did not necessarily result in high prevalence of psychological symptoms.

Nevertheless, the school- and country-level prevalence of bullying may moderate the effects of bullying on individuals’ well-being. A study by Brickman and Bulman [37] was the first to show that individuals who are experiencing difficulties in their lives, become more stressed when they compare themselves with others who are doing well. On the other hand, people may find it encouraging when they come across others who are facing similar or even worse problems than they are. The latter comparison might put ones’ own situation in perspective [38]. In the context of the current study, we could argue that the social comparison of bully victims may be more favourable in classes and schools where they see that they are not alone in having to deal with this problem. Similarly, if bullying is infrequent, you may well be the only kid in your class that is a victim. This might also apply on a country-level and thus give a different perspective on Due et al.’s [3] international comparison.

Social comparison, i.e., comparing oneself, one’s status or one’s situation with that of others, is deeply ingrained in the human psyche. We frequently engage in this process to obtain information about ourselves, and usually do so without conscious effort or intention. It has been argued that significant life events may cause changes in the nature and amount of social comparison of an individual. The need to know how one is doing is also likely to increase during stressful times [38]. The turbulence of adolescence combined with the agony of bully victimization is certainly going to increase stress levels and preoccupation with social cues. 

The hypothesis put forth in the current paper is that being the victim of bullying in an environment where bullying is rare, is more detrimental to ones’ life satisfaction than if victimization is more prevalent. The conceptual framework of social comparison also predicts that being bullied in an environment where many other students are also experiencing it, may provide emotional, physical and intellectual consolation for victims. In this sense, bullying may become a greater problem for individual students as it becomes less of a problem for schools and society in general. 

## 2. Materials and Methods 

This paper examines the effects of bullying on life satisfaction among children in 35 Western, industrialized countries (n = 183,736). 

Analyses were based on data from the 2005/2006 Health Behaviour in School-aged Children study (available at hbsc.org), a World Health Organization collaborative cross-national study [39]. The international standard questionnaire consists of core questions used in all participating countries and additional focus questions that allow participating countries to emphasize areas of national interest. The measures in the current study were used in 35 Western, industrialized countries (Austria, Belgium, Bulgaria, Canada, Croatia, Czech Republic, Denmark, Estonia, Finland, France, Germany, Greece, Greenland, Hungary, Iceland, Ireland, Italy, Latvia, Lithuania, Luxembourg, Macedonia, Netherlands, Norway, Poland, Portugal, Romania, Russia, Slovenia, Spain, Sweden, Switzerland, Turkey, Ukraine, the United Kingdom, and the United States). Ethical approval for each national survey was obtained according to the relevant regulations in each country.

A nationally representative random sample of 11-, 13- and 15-year-old school children was drawn with the recommended minimum sample size of 1536 students per age group in each country. About 80% of the schools contacted allowed the survey to take place in selected classes, and refusals at the student level were very rare. Listwise deletion of missing cases reduced the total sample size by 6.4%, leading to a net sample of 183,736 students. 

Descriptive statistics of individual-level, school-level and country-level predictors of life satisfaction are shown in Table 1. The dependent variable of life satisfaction is measured by Cantril’s [20] classic measure, asking respondents to indicate where they feel that they stand at the moment or based on a visual representation of a ladder with 0 representing the worst possible life and 10 the best possible life. This measure has been extensively used in prior studies of life satisfaction for both adults e.g., [21,22,23,24] and children [25,26,27,28,35].

Life satisfaction as measured by Cantril’s [20] ladder from the worst possible life to the best possible life, is an efficient global measure with high construct validity across diverse socio-demographic factors such as age, gender, height, income, marital status, employment and religion e.g., [21,22,23]. A study by Levin and Currie [40] found that across samples of 11–15-year-old pupils, the Cantril Ladder showed good reliability, and among 11-year olds, even better than other popular tests used for adolescents. It also demonstrated good convergent validity with other emotional well-being measures, perceived health and subjective health.

Studies conducted in several countries in recent years have demonstrated that Cantril’s [20] measure of life satisfaction is associated with various health behaviours among children, including binge drinking in Wales [25], addictive behaviours in the Netherlands [26], food poverty in Ireland [27], lack of physical activity and screen-based media use in Canada and the United States [28], and family structure in North-America and Europe [35]. 

The HBSC questionnaire includes an adapted version of the Cantril Ladder similar to the original version but leaves out the opening section where people note their idea of the best and worst possible life and contains only a ladder relating to the present [40].

Olweus [41] developed questions to measure individual-level bully victimization, like “How often have you been bullied at school in the past couple of months?” The following definition of bullying preceded the question: “We say a student is being bullied when another student, or group of students, say or do nasty and unpleasant things to him or her. It is also bullying when a student is teased repeatedly in a way he or she does not like or when he or she is deliberately left out of things. But it is not bullying when two students about the same strength or power argue or fight. It is also not bullying when a student is teased in a friendly and playful way.” Response categories ranged from 1: “I have not been bullied at school in the past couple of months” to 5: “several times a week”. Responses were recoded as an approximation of monthly prevalence (0 = never, 8 = at least 8 times a month). School-level and country-level bullying was measured as the mean of individual-level bullying in each school and country.

The following data analysis is based on multi-level modelling techniques [42,43] and carried out with *HLM 6.0* software (Scientific Software International, Chicago, IL, USA) [44]. This methodology allows us to address several important theoretical and conceptual issues. Hierarchical linear regression extends the general multiple regression model and allows estimating individual-level models of the effects of exteriority and constraint and other indicators on various outcomes as:

$$Y_{ijk}=\beta_{0jk}+\sum_{q=1}^{Q}\beta_{qjk}X_{qijk}+r_{ijk}s$$

where $Y_{ijk}$ is the outcome of student *i* in school *j* within country *k*, $\beta_{0jk}$ is the individual-level intercept for each school *j* within each country *k*, $\beta_{qjk}$ (*q* = 1, 2,..., *Q*) are individual-level slopes for each school *j* within each country *k*, $X_{qijk}$ is the *q*th individual-level predictor for student *i* in school *j* in country *k* and $r_{ijk}$ is the individual-level error term see [44]. This extends the general regression model by allowing the estimation of variable intercept models of the effects of school-level and country-level predictors on individual-level adolescent outcomes, as well as allowing the estimation of variable slopes for individual-level predictors across countries. Each of the individual-level coefficients $\beta_{qjk}$ can thus be modelled as an outcome variable moderated by the school and country level. All individual-level predictors are centred at the grand mean in the following analysis.

The following analysis considers a three-level model of life satisfaction. The first level includes the individual measures of gender, age and exposure to bullying. This allows us to estimate the intercept for life satisfaction across all countries and the individual-level association between exposure to bullying and life satisfaction, controlling for gender and age. 

The second level includes school levels of exposure to bully victimization as a moderator of both the intercept for life satisfaction and the slope between bullying and life satisfaction. This allows us to estimate the effect of bullying prevalence in different schools on both baseline life satisfaction and the strength of the individual-level association between bullying and life satisfaction.

The third level includes country levels of exposure to bully victimization as a moderator of both the intercept for life satisfaction and the slope between bullying and life satisfaction. This allows us to estimate the effect of bullying prevalence in different countries on both baseline life satisfaction and the strength of the individual-level association between bullying and life satisfaction.

No statistically significant interactions were found between gender and other predictors in this study.

## 3. Results

Table 2 shows the multi-level analysis for life satisfaction.

The results show that the intercept for life satisfaction varies significantly between schools and countries. Males report significantly higher life satisfaction than females, and life satisfaction generally decreases with age, but these effects also differ significantly between schools and countries. As expected, life satisfaction decreases with increased prevalence of bullying on the individual level, but the strength of this association is significantly different across schools and countries.

The second part of Table 2 shows that individual life satisfaction decreases significantly as the school prevalence of bully victimization increases, net of individual experiences of being bullied. However, the country prevalence of bullying is not significantly associated with individual differences in life satisfaction, net of such individual-level and school-level effects.

Finally, the third part of Table 2 shows that the school level and the country level of bully victimization significantly moderates the individual-level association between bullying and life satisfaction. The negative slope between individual experiences of victimization and individual life satisfaction is significantly decreased as school- and country-levels of bullying increase. 

**Figure 1:**
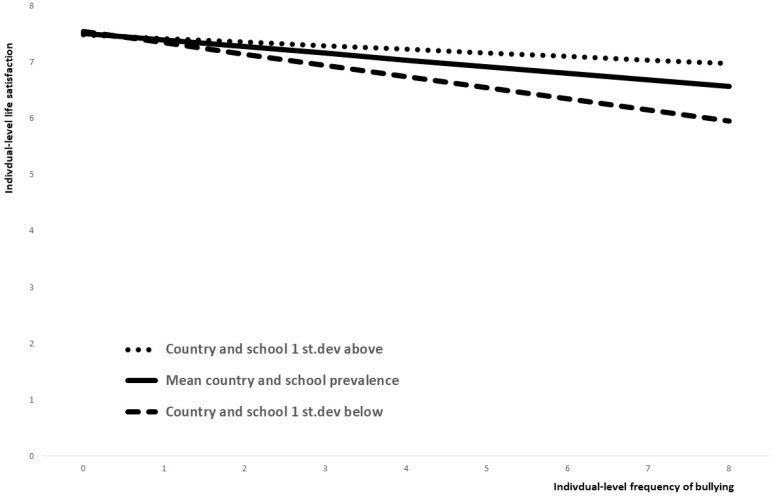
Example of varying intercepts and slopes.

The variable intercepts and slopes represent a whole range of different regression equations according to country and school levels of bullying. Figure 1 shows three examples of the differing association of individual-level bullying and life satisfaction. The middle line shows the estimated association for students in a country and a school with an average prevalence of bullying. The top line shows the estimated association for students where both the country and the school are one standard deviation above the average level, and the bottom line the estimated association where both the country and the school are one standard deviation below the average level of bullying.

Overall, bullying has a negative effect on life satisfaction, but this association is stronger in countries and schools where bullying is less prevalent. Students that are never bullied on average score about 7, 5 on life satisfaction, with a slightly but statistically significantly higher life satisfaction in schools where bullying is less prevalent. 

In this example, a student who is bullied twice a week has an estimated life satisfaction of 6.6 if country and school levels of bullying are average. However, the estimated life satisfaction is 7.0 if both country and school levels of bullying are one standard deviation above the average and 5.9 if both country and school levels of bullying are one standard deviation below the average.

## 4. Discussion

Our hypothesis, that being the victim of bullying in an environment where bullying is rare is more detrimental to ones’ life satisfaction than if victimization is more prevalent, is supported by the analysis. Our results may explain the lack of association in between-country differences in bully victimization and symptom prevalence as described by Due et al. [3]. The results indicate that when bullying takes place in an environment where it is otherwise infrequent, it can exacerbate the victim’s negative feelings as a result. In other words, it is worse being bullied when you are one of a few victims than when bullying is relatively commonplace. Being the only one bullied in your class is therefore likely to cause much more harm to your life satisfaction than, for example, if it also happens to a third of your classmates.

Adolescents report their life satisfaction in accordance with their own standards [18], which they will set by comparing themselves to their peers. According to the multiple discrepancy theory of satisfaction [45], individuals are constantly comparing themselves to certain standards, including people in their environment. Amongst other things, people base satisfaction judgments on discrepancies between their own situation and the situation of those around them, where negative comparison will lead to negative satisfaction. Applying his theory Michalos [46] found for example that college students’ perception of how their health status compared with their peers was the strongest predictor of their overall satisfaction with their own health. It is reasonable to suggest that similar social comparison mechanism is at least partly responsible for the results we see in the current study.

Peer relationships matter very much to adolescents. This, coupled with the fact that this is on average the loneliest time of a person’s life [47], makes social isolation a key mental health issue for this age group. Adolescents worry excessively about their status within their peer group and are therefore constantly comparing themselves to others. Experiencing that you are the only one being victimized will naturally compound the effects of the bullying per se.

## 5. Conclusions

In summary, we have identified prevalence of bullying in the students’ surroundings as a mediating variable explaining the relationship between bully victimization and life satisfaction. These results are a stern reminder that although it is possible to achieve great results in reducing the numbers of bullied adolescents through prevention and intervention, it is very important to stay focused on the problem even though fewer victims are involved. The problem of infrequent bullying is that favourable percentages may conceal real suffering. 

## Figures and Tables

**Table 1:** Descriptive statistics for country-level and individual-level predictors of life satisfaction for 11- to 15-year-old students in 35 countries, HBSC 2005–2006.

	Min	Max	M	SD
**Country-Level (n = 35)**				
Country-level bullying	0.35	1.85	0.92	0.40
**School-Level (n = 6726)**				
School-level bullying	0.00	8.00	0.91	0.68
**Individual-Level (n = 183,736)**				
Gender (male)	0.00	1.00	0.49	0.51
Age (in years)	10.50	16.58	13.60	1.65
Individual-level bullying	0.00	8.00	0.88	1.80
**Dependent Variable (n = 183,736)**				
Life satisfaction	0.00	10.00	7.56	1.92

**Table 2:** Multi-level analysis of life satisfaction and bullying for 11- to 15-year-old students in 35 countries, HBSC 2005–2006.

**Individual-Level Predictors of Life Satisfaction**				
	**Coeff.**	**S.E.**	**School Variance**	**Country Variance**
Intercept	7.55 ***	0.05	0.057 ***	0.079 ***
Gender	0.21 ***	0.02	0.095 ***	0.010 ***
Age	−0.23 ***	0.01	0.008 ***	0.003 ***
Bullying	−0.21 ***	0.01	0.019 ***	0.003 ***
**Direct Effects of School- and Country-Level Bullying on Individual-Level Life Satisfaction**				
	**Coeff.**	**S.E.**		
School prevalence of bullying	−0.04 **	0.01		
Country prevalence of bullying	−0.02	0.14		
**Moderating Effects of School- and Country-Level Bullying on the Individual-Level Association between Having Been Bullied and Life Satisfaction**				
	**Coeff.**	**S.E.**		
School prevalence	0.05 ***	0.01		
Country prevalence	0.05 *	0.02		

* *p* < 0.05, ** *p* < 0.01, *** *p* < 0.001.
